# Cerebral gray matter volume reduction in subcortical vascular mild cognitive impairment patients and subcortical vascular dementia patients, and its relation with cognitive deficits

**DOI:** 10.1002/brb3.745

**Published:** 2017-06-23

**Authors:** Maoyu Li, Yao Meng, Minzhong Wang, Shuang Yang, Hui Wu, Bin Zhao, Guangbin Wang

**Affiliations:** ^1^ Department of Neurology Shandong Provincial Hospital Affiliated to Shandong University Jinan Shandong China; ^2^ Department of Magnetic Resonance Imaging Shandong Medical Imaging Research Institute Affiliated to Shandong University Jinan Shandong China

**Keywords:** cerebral GM volume reduction, cognitive deficits, SVaD, svMCI

## Abstract

**Introduction:**

Subcortical vascular mild cognitive impairment (svMCI) is the predementia stage of subcortical vascular dementia (SVaD). The aim of this research is to explore and compare cerebral gray matter (GM) volume reduction in svMCI patients and SVaD patients, and to investigate the relationship between cerebral GM volume reduction and cognitive deficits.

**Methods:**

Thirty one svMCI patients, 29 SVaD patients, and 31 healthy controls were recruited in our research. They conducted neuropsychological tests and brain structural magnetic resonance imaging (MRI) examination. To detect cerebral GM volume reduction in svMCI patients and SVaD patients, we used statistical parametric mapping 8‐voxel‐based morphometry 8 (SPM8‐VBM8) method to analyze MRI data. To detect the relationship between cerebral GM volume reduction and cognitive deficits, multiple linear regression analysis was used.

**Results:**

Compared with healthy controls, svMCI patients showed cerebral GM volume reduction in hippocampus and parahippocampal gyrus, insula and superior temporal gyrus. Compared with healthy controls, SVaD patients exhibited more atrophy which encompasses all of these areas plus anterior and middle cingulate, inferior temporal gyrus, orbitofrontal cortex, and superior frontal gyrus. In svMCI patients, cerebral GM volume reduction correlated with memory loss, attention dysfunction, and language dysfunction; in SVaD patients, besides those cognitive deficits, cerebral GM volume reduction correlated with more cognitive impairments, including executive dysfunction, neuropsychiatric symptom, and depression.

**Conclusions:**

Our findings prove that both svMCI patients and SVaD patients exhibit cerebral GM volume reduction and there may exist a hierarchy between svMCI and SVaD, and cerebral GM volume reduction in both svMCI patients and SVaD patients correlates with cognitive deficits, which can help us understand the mechanism of cognitive impairments in svMCI patients and SVaD patients, and diagnose SVaD at its early stage.

## 1 INTRODUCTION

Vascular dementia (VaD) is considered to be the second most common cause of dementia following Alzheimer's disease (AD) (Korczyn, Vakhapova, & Grinberg, 2012; O'Brien & Thomas, 2015). One type of VaD, called subcortical vascular dementia (SVaD), results from small vessel disease, and causes cognitive deficits in elderly people (Román, Erkinjuntti, Wallin, Pantoni, & Chui, 2002). SVaD can be further classified into multiple lacunar infarctions subtype and Binswanger's disease subtype, which has its respective characteristics (Tomimoto, 2011). The former is characterized by lacunar infarctions and the latter is characterized by diffuse white matter lesions (Tomimoto, 2011). SVaD patients exhibit cognitive impairments in attention, executive function, and retrieval of memory (Baker et al., 2012). Besides cognitive impairments, SVaD patients also present clinical symptoms, such as urinary incontinence, pseudobulbar palsy, dysarthria, dysphagia, and hemiparesis (Roh & Lee, 2014), which may disable patients and cause great trouble to patients’ life. Thus, it is extremely important to diagnose and treat SVaD at its early stage.

The damage of cognition in mild cognitive impairment (MCI) patients is mild, and it will not affect patient's daily life. The predementia stage of SVaD (Frisoni, Galluzzi, Bresciani, Zanetti, & Geroldi, 2002; Galluzzi, Sheu, Zanetti, & Frisoni, 2005), called subcortical vascular mild cognitive impairment (svMCI), is also caused by cerebral small vessel abnormalities. Detecting svMCI patients on magnetic resonance imaging (MRI), multiple lacunar infarctions and white matter hyperintensities (WMH) can be found (Noh et al., 2014; Yi et al., 2012).

The pathogenesis of svMCI and SVaD remains unclear, which leads to the hypothesis that the disruption of frontal‐subcortical loops and long association fibers may play a significant part (Helena C. Chui, 2007). Nevertheless, multiple lacunar infarctions or WMH, which represent cerebral small vessel disease, is limited to diagnose dementia because some elderly people, with similar subcortical vascular lesions, have no cognitive deficits (Pascual et al., 2010). Thus, some authors pointed out that multiple lacunar infarctions and WMH are the characteristics of cerebral small vessel disease, but have no relation with cognitive impairments (Sabri et al., 1998). Previous studies have found cerebral gray matter (GM) volume reduction in svMCI patients and SVaD patients (Li, Du, Zheng, & Wang, 2011; Liu et al., 2014; Seo et al., 2010; Yi et al., 2012), involving not only association cortices, but also primary cortices and subcortical GM nucleus, which is different from the atrophy pattern in AD. As there are many regions in cerebral GM that are responsible for normal cognitive functions, we speculate that it is cerebral GM volume reduction that causes cognitive impairments, such as executive dysfunction, memory loss, and language dysfunction. Nevertheless, few researches have investigated the relationship between cerebral GM volume reduction and the subfield of cognitive deficits in svMCI patients and SVaD patients.

The aim of this research is to explore and compare cerebral GM volume reduction in svMCI and SVaD patients, and to investigate the relationship between cerebral GM volume reduction and cognitive deficits, which can help us understand the mechanism of cognitive impairments in svMCI and SVaD patients, and diagnose SVaD at its early stage.

## 2 MATERIALS AND METHODS

### 2.1 Ethics statement

All procedures of our research were performed in accordance with the ethical standards of the responsible committee on human experimentation and with the Helsinki Declaration of 1975, as revised in 2008. This study was reviewed and approved by the Ethics Committee in Shandong Provincial Hospital Affiliated to Shandong University. Before the study, all participants gave their written informed consent.

### 2.2 Subjects

Sixty right‐handed people including 31 svMCI patients and 29 SVaD patients, who have visited the neurologic clinic in Shandong Provincial Hospital from March 2015 to May 2016, were included in this research. The diagnosis of SVaD was on the basis of Erkinjuntti's brain imaging criteria (Erkinjuntti et al., 2000) and ADDTC (Alzheimer's Disease Diagnostic and Treatment Centers) criteria (H. C. Chui et al., 1992) for probable or possible VaD. The diagnosis of svMCI was on the basis of Erkinjuntti's brain imaging criteria (Erkinjuntti et al., 2000), Peterson's criteria (Petersen, 2004) with the following modifications which were previously described (Noh et al., 2014): (i) subjective cognitive complaints; (ii) objective decline of cognition below 1.5 *SD* of norms on standardized neuropsychological tests; (iii) normal or near normal activity of daily living; (iv) no dementia; (5) CDR score = 0.5; (6) focal neurological symptoms. A total of 31 age‐ and sex‐matched people with normal cognition were recruited as healthy controls from the department of physical examination center in Shandong Provincial Hospital. All healthy controls were free of cerebral infarction, hemorrhage, or other disease that may affect cognitive functions.

All patients and controls have undergone neurological, neuropsychological, and neuroimaging evaluation. Cognitive performances were assessed at neuropsychological measuring room in Shandong Provincial Hospital. Neuropsychological tests include Mini Mental State Examination (MMSE), Montreal Cognitive Assessment (MoCA), Activity of Daily Living Scale (ADL), Clinical Dementia Rating Scale (CDR), Hachinski ischemic scale (HIS), Neuropsychiatric Inventory (NPI), Geriatric Depression Scale (GDS), Trail Making Test (TMT), Auditory Verbal Learning Test (AVLT), Symbol Digit Modalities Test (SDMT), and Verbal Fluency Test (VFT). To ensure that SVaD patients with mild to moderate dementia were included in this study, SVaD patients whose CDR score˃2 were excluded. Also, for the purpose of excluding mixed dementia, svMCI patients and SVaD patients whose HIS score<7 were excluded from this study.

Exclusion criteria for recruitment in the study were as follows: (i) patients who have cortical infarction, embolic infarction, or acute infarction; (ii) ˃50% stenosis of extracranial carotid artery, vertebral artery, or intracranial artery; (iii) neurological diseases, systemic autoimmune disease, or other major medical disease which may cause cognitive impairments; (iv) major psychiatric disorder, head trauma, or drug abuse.

### 2.3 Clinical imaging data analysis

The imaging markers of svMCI and SVaD include WMH and lacunes according to Erkinjuntti's brain imaging criteria (Erkinjuntti et al., 2000). WMH were defined as high‐signal intensity area on T2‐weighted imaging (T2WI) or fluid‐attenuated inversion recovery (FLAIR) imaging, and graded according to Fazekas scale (Fazekas, Chawluk, Alavi, Hurtig, & Zimmerman, 1987). Lacunes were defined as 3–15 mm abnormalities with signal equivalent to cerebral spinal fluid (CSF) on T2WI/Flair imaging. The Erkinjuntti's brain imaging criteria include “white matter cases” which mean severe periventricular and deep WMH and at least one lacune in deep GM or “lacunar cases” which mean multiple lacunes (˃5) in the deep GM and at least moderate WMH (Erkinjuntti et al., 2000).

### 2.4 MRI acquisition

Data were collected on a MAGNETOM Skyra 3‐Tesla scanner (Siemens Healthcare GmbH, Erlangen, Germany) with a 32‐channel head coil. T1‐weighted magnetization‐prepared rapid gradient echo (T1‐MPRAGE) axial images were collected, and parameters were as follows: repetition time = 1,900 ms, inversion time = 900 ms, echo time = 2.52 ms, matrix = 256 × 256, flip angle = 9°, no gap, thickness = 1.0 mm, 176 slices, and voxel size = 1 × 1 × 1 mm^3^. Conventional T1‐weighted imaging (T1WI), T2WI, FLAIR imaging, and diffusion‐weighted imaging (DWI) were also collected to inspect structural abnormalities.

### 2.5 Image processing

Basic VBM8 (http://dbm.neuro.uni-jena.de/vbm/, RRID: SCR_014196) analysis comprises spatial preprocessing and statistical analysis. Spatial preprocessing used SPM8 (http://www.fil.ion.ucl.ac.uk/spm, RRID: SCR_007037) based on MATLAB (http://www.mathworks.com/products/matlab/, RRID: SCR_001622). T1‐MPRAGE images were normalized to MNI template and segmented into GM, white matter (WM), and CSF volume maps. The segmented maps underwent data quality checking and smoothing (Gaussian kernel, 8 mm full width at half maximum).

### 2.6 Statistical analysis

#### 2.6.1 Statistical analysis of demographic data, MRI findings, and neuropsychological test results

We used SPSS software 17.0 version package to perform statistical analysis of demographic data, MRI findings, and neuropsychological test results. Chi‐square test was used to compare categorical demographic variables. One way analysis of variance was used for comparison of continuous variables, followed by post hoc analysis using Student‐Neuman‐Keuls (SNK) test (comparison of CDR, ADL, and HIS was not included in this study). Statistical significance was threshold at *p *<* *.05.

#### 2.6.2 Voxel‐based morphological analysis of cerebral GM volume

Segmented cerebral GM maps were statistically analyzed by SPM8 (http://www.fil.ion.ucl.ac.uk/spm, RRID: SCR_007037). The difference of cerebral GM volume among subject groups were explored by the full factorial model (one‐way analysis of covariance), followed by post hoc analysis. Age, gender, educational level, and total brain volume were used as covariates. The *t* values of resulting cerebral GM volume parametric maps were thresholded at *p* = .001 at voxel level, and *p *=* *.05 corrected for multiple comparisons based on FDR (False Discovery Rate) at cluster level. Anatomic labeling of cluster localizations and statistical maps were acquired by xjView software (http://www.alivelearn.net/xjview8/, RRID: SCR_008642).

#### 2.6.3 Analysis of correlation between neuropsychological test results and the mean cerebral GM volume

For the purpose of exploring whether neuropsychological test results (which were significantly different between subject groups) (CDR, ADL, and HIS were not included) correlate with the mean cerebral GM volume (of the area where cerebral GM volume was significantly different between subject groups), multiple linear regression analysis was used. Age, gender, and educational level were controlled. The mean cerebral GM volume (of the area where cerebral GM volume was significantly different between subject groups) was used as independent variables, and neuropsychological test results were used as dependent variables.

## 3 RESULTS

### 3.1 Demographic data and MRI findings

The demographic data and MRI findings of healthy controls, svMCI patients, and SVaD patients were shown in Table 1. These three groups did not have significant difference in age, gender, educational level, or total brain volume (*p *<* *.05). Significant difference was found in numbers of lacunes and WMH volume among these three groups (*p *<* *.05). Number of lacunes and WMH volume in svMCI patients and SVaD patients were significantly higher than healthy controls (*p *<* *.05). When comparing number of lacunes and WMH volume between svMCI and SVaD patients, no significant difference was found (*p *˃ .05). Significant difference was found in total cerebral GM volume among these three groups (*p *<* *.05). Total cerebral GM volume in svMCI patients and SVaD patients was significantly smaller than that in healthy controls (*p *<* *.05), and total cerebral GM volume in SVaD patients was significantly smaller than that in svMCI patients (*p *<* *.05).

**Table 1:** Demographic data and MRI findings of participants

	SVaD	svMCI	HC
Number	29	31	31
Age (years)	64.4 (6.8)	63.2 (6.4)	62.7 (6.9)
Male: Female	16:13	14:17	16:15
Education (years)	6.5 (3.8)	7.1 (3.3)	7.5 (3.8)
WMH volume (cm^3^)	29.8 (7.3)*	28.1 (6.5)*	3.2 (0.8)
Lacunes (n)	10.4 (2.5)*	9.5 (2.6)*	1.1 (0.8)
Total cerebral GM volume (cm^3^)	518.4 (44.0)*, #	559.2 (72.7)*	620.0 (34.5)
Total brain volume (cm^3^)	1407.2 (107.4)	1399.3 (183.4)	1454.3 (146.6)

Data were presented as the range of min‐max (mean ± *SD*). HC, healthy controls; svMCI, subcortical vascular cognitive impairment; SVaD, subcortical vascular dementia. *p <* *.05 between HC and svMCI or SVaD; #*p *<* *.05 between svMCI and SVaD.

### 3.2 Neuropsychological test results

The neuropsychological test results of healthy controls, svMCI patients, and SVaD patients are shown in Table 2. These three groups had significant difference in scores of MMSE, MoCA, NPI, GDS, and of executive function (TMT‐A, TMT‐B), memory (AVLT‐immediate recall, AVLT‐delayed recall, AVLT‐recognition), attention (SDMT), and language (VFT) (*p *<* *.05). Post hoc analysis proved that compared with healthy controls, svMCI patients performed worse in scores of MoCA, GDS, and of executive function (TMT‐B), memory (AVLT‐immediate recall, AVLT‐delayed recall, AVLT‐recognition), attention (SDMT), and language (VFT) (*p *<* *.05). Compared with healthy controls, SVaD patients performed worse in all neuropsychological test results which were performed in our study (CDR, ADL, and HIS were not included) (*p *<* *.05). At the same time, post hoc analysis revealed that compared with svMCI patients, SVaD patients showed poorer performance in scores of MMSE, MoCA, NPI, GDS, and of executive function (TMT‐A, TMT‐B), attention (SDMT), and language (VFT) (*p *<* *.05).

**Table 2:** Neuropsychological test results of participants

	SVaD	svMCI	HC
MMSE	16.1 (3.7)*, #	25.4 (2.5)	26.2 (2.1)
MoCA	14.0 (3.5)*, #	23.7 (2.7)*	27.1 (1.8)
CDR	1.4 (0.6)	0.5	0
ADL (Barthel Index)	44.5 (12.3)	77.3 (8.6)	–
HIS	9.9 (1.8)	9.5 (1.7)	–
NPI	14.8 (3.9)*, #	7.6 (3.4)	6.1 (3.0)
GDS	8.2 (2.1)*, #	6.1 (2.2)*	3.8 (1.3)
Executive function			
TMT‐A (s)	76.5 (21.3)*, #	63.5 (16.0)	56.9 (18.2)
TMT‐B (s)	203.5 (40.5)*, #	177.8 (38.0)*	151.2 (33.0)
Memory			
AVLT‐immediate recall	4.0 (1.3)*	4.8 (2.2)*	7.9 (2.3)
AVLT‐delayed recall	4.5 (1.5)*	5.1 (2.1)*	9.1 (2.8)
AVLT‐recognition	7.1 (2.0)*	8.2 (2.8)*	12.4 (3.5)
Attention			
SDMT	21.8 (6.0)*, #	26.0 (6.0)*	30.3 (6.6)
Language			
VFT	13.6 (3.6)*, #	16.7 (4.8)*	20.9 (5.8)

Data were presented as the range of min‐max (mean ± *SD*). HC, healthy controls; svMCI, subcortical vascular cognitive impairment; SVaD, subcortical vascular dementia; MMSE, Mini Mental State Examination; MoCA, Montreal Cognitive Assessment; CDR, Clinical Dementia Rating Scale; ADL, Activity of Daily Living Scale; HIS, Hachinski Ischemic Scale; NPI, Neuropsychiatric Inventory; GDS, Geriatric Depression Scale; TMT, Trail Making Test; AVLT, Auditory Verbal Learning Test; SDMT, Symbol Digit Modalities Test; VFT, Verbal Fluency Test. *p <* *.05 between HC and svMCI or SVaD; #*p *<* *.05 between svMCI and SVaD.

### 3.3 Voxel‐based analysis of cerebral GM volume

**Figure 1:**
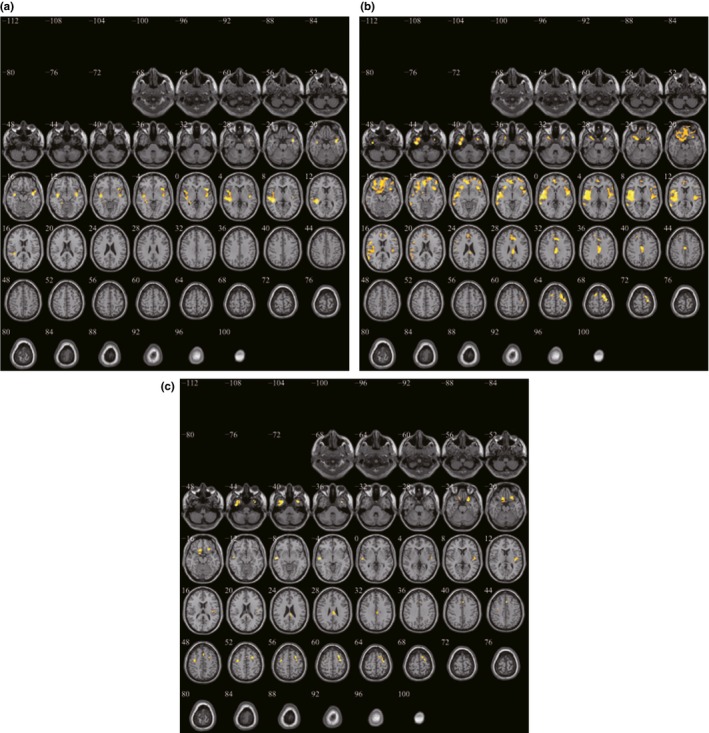
Detected by MRI using SPM8‐VBM8 method, the area of cerebral GM volume reduction in (a) svMCI patients compared with healthy controls, (b) SVaD patients compared with healthy controls, and (c) SVaD patients compared with svMCI patients are shown in yellow color at axial position (FDR‐corrected *p *<* *.05 at cluster level). Age, gender, and educational level were used as covariates. Lighter area represents region with a greater statistical difference

Compared with healthy controls, svMCI patients exhibited significant reduction in cerebral GM volume on whole‐brain VBM analysis, including bilateral hippocampus and parahippocampal gyrus, bilateral insula and superior temporal gyrus (FDR‐corrected *p *<* *.05 at cluster level) (Figure 1a and Table 3). Compared with healthy controls, SVaD patients exhibited significant reduction in cerebral GM volume, including bilateral hippocampus and parahippocampal gyrus, dorsolateral temporal lobe cortex, insula, superior frontal gyrus, and orbitofrontal cortex, as well as anterior and middle cingulate (FDR‐corrected *p *<* *.05 at cluster level) (Figure 1b and Table 4). Furthermore, compared with svMCI patients, significant reduction in cerebral GM volume was located at bilateral middle cingulate, inferior temporal gyrus, orbitofrontal cortex, and superior frontal gyrus, as well as left middle temporal gyrus and right insula in SVaD patients (FDR‐corrected *p *<* *.05 at cluster level) (Figure 1c and Table 5). Compared with healthy controls, no significant cerebral GM volume increases were found in svMCI patients and SVaD patients (FDR‐corrected *p *˃ .05 at cluster level).

**Table 3:** Local maximums of significant clusters showing cerebral GM volume reduction in svMCI patients compared with healthy controls (FDR‐corrected *p *<* *.05 at cluster level)

Region	MNI Coordinates			Voxel numbers	*T* value
	X	Y	Z		
Right insula	38	−5	−17	1,029	7.59
Left superior temporal gyrus	−41	−31	12	1,637	6.97
Right superior temporal gyrus	42	−3	−14	336	6.85
Right hippocampus and parahippocampal gyrus	14	2	−18	479	6.06
Left hippocampus and parahippocampal gyrus	−35	−22	−2	425	5.18
Left insula	−42	−14	8	807	5.06

**Table 4:** Local maximums of significant clusters showing cerebral GM volume reduction in SVaD patients compared with healthy controls (FDR‐corrected *p *<* *.05 at cluster level)

Region	MNI Coordinates			Voxel numbers	*T* value
	X	Y	Z		
Left dorsolateral temporal lobe cortex	−54	5	−5	3,808	7.95
Right superior frontal gyrus	26	0	67	661	7.37
Bilateral anterior cingulate	−2	16	17	1,302	7.03
Right hippocampus and parahippocampal gyrus	24	−3	−27	379	6.95
Left insula	−42	−12	9	1,846	6.82
Right insula	49	2	−2	1,838	6.77
Left orbitofrontal cortex	−15	24	−22	2,692	6.63
Bilateral middle cingulate	5	−17	36	1,464	6.48
Right dorsolateral temporal lobe cortex	47	−3	−24	1,215	6.11
Right orbitofrontal cortex	13	41	−3	1,971	5.97
Left superior frontal gyrus	−15	11	68	291	5.93
Left hippocampus and parahippocampal gyrus	−40	−29	3	392	5.35

**Table 5:** Local maximums of significant clusters showing cerebral GM volume reduction in SVaD patients compared with svMCI patients (FDR‐corrected *p *<* *.05 at cluster level)

Region	MNI Coordinates			Voxel numbers	*T* value
	X	Y	Z		
Left middle temporal gyrus	−56	−14	−6	367	7.22
Right superior frontal gyrus	30	−5	60	619	6.56
Left inferior temporal gyrus	−32	3	−39	402	6.48
Right inferior temporal gyrus	35	6	−39	277	6.33
Left orbitofrontal cortex	−17	21	−17	257	5.92
Right orbitofrontal cortex	21	12	−26	464	5.83
Left superior frontal gyrus	−38	−6	53	223	5.51
Bilateral middle cingulate	3	−26	27	337	5.38
Right insula	48	−12	9	503	5.26

### 3.4 Correlation between neuropsychological test results and the mean cerebral GM volume

There were significant correlation between neuropsychological test results (which were significantly different between subject groups) (CDR, ADL and HIS were not included) and the mean cerebral GM volume (of the area where cerebral GM volume was significantly different between subject groups). In healthy controls versus svMCI patients group, the mean cerebral GM volume (of the area where cerebral GM volume was significantly different between healthy controls and svMCI patients) significantly correlated with scores of MoCA, and of memory (AVLT‐immediate recall, AVLT‐delayed recall, AVLT‐recognition), attention (SDMT), and language (VFT) (*p *<* *.05) (Table 6). In healthy controls versus SVaD patients group, the mean cerebral GM volume (of the area where cerebral GM volume was significantly different between healthy controls and SVaD patients) significantly correlated with all neuropsychological test results which were performed in our study (CDR, ADL, and HIS were not included) (*p *<* *.05) (Table 6).

**Table 6:** Correlation between neuropsychological test results and the mean cerebral GM volume (of the area where cerebral GM volume was significantly different between subject groups)

	HC and svMCI			HC and SVaD		
	*B* (*SE*)	*t*	*p*	*B* (*SE*)	*t*	*p*
MMSE	–	–	–	3.70 (0.93)	3.97	<.001
MoCA	2.44 (0.59)	4.13	<.001	2.94 (0.96)	3.06	.005
NPI	–	–	–	−3.34 (1.08)	−3.08	.005
GDS	−0.56 (0.59)	−0.94	.357	−2.79 (0.38)	−7.25	<.001
Executive function						
TMT‐A (s)	–	–	–	−20.80 (5.46)	−3.81	.001
TMT‐B (s)	−4.99 (10.60)	−0.47	.641	−47.41 (9.14)	−5.19	<.001
Memory						
AVLT‐immediate recall	2.41 (0.44)	5.46	<.001	1.63 (0.29)	5.57	<.001
AVLT‐delayed recall	1.43 (0.51)	2.78	.010	1.40 (0.41)	3.46	.002
AVLT‐recognition	2.30 (0.67)	3.42	.002	2.58 (0.38)	6.80	<.001
Attention						
SDMT	4.97 (1.40)	3.55	.001	5.25 (1.62)	3.23	.003
Language						
VFT	3.09 (1.20)	2.57	.015	2.71 (1.03)	2.65	.013

HC, healthy controls; svMCI, subcortical vascular cognitive impairment; SVaD, subcortical vascular dementia; MMSE, Mini Mental State Examination; MoCA, Montreal Cognitive Assessment; NPI, Neuropsychiatric Inventory; GDS, Geriatric Depression Scale; TMT, Trail Making Test; AVLT, Auditory Verbal Learning Test; SDMT, Symbol Digit Modalities Test; VFT, Verbal Fluency Test.

## 4 DISCUSSION

Using SPM8‐VBM8 method to analyze MRI data, we found that both svMCI patients and SVaD patients exhibited cerebral GM volume reduction and there existed a hierarchy between svMCI and SVaD. Compared with healthy controls, the area of cerebral GM volume reduction in svMCI patients (e.g., hippocampus and parahippocampal gyrus, insula, and superior temporal gyrus) was slightly smaller than previous researches (Seo et al., 2010; Yi et al., 2012), probably due to the difference of included criteria. SVaD patients showed widespread cerebral GM volume reduction, involving frontal lobe cortices, temporal lobe cortices, insular lobe cortices etc., which was basically in accordance with previous studies (Li et al., 2011; Liu et al., 2014; Seo et al., 2010). At the same time, it has been proven in our research that the mean cerebral GM volume (of the area where cerebral GM volume was significantly different between healthy controls and svMCI patients, healthy controls and SVaD patients) significantly correlated with neuropsychological test results in svMCI patients and SVaD patients, respectively, which is the first study to certify the relationship between the subfield of cognitive deficits and cerebral GM volume reduction in svMCI patients and SVaD patients.

Hippocampus might be the most vulnerable region to ischemic damage (Kirino & Sano, 1984; Pulsinelli, Brierley, & Plum, 1982). As hippocampus and its related structure are involved in memory consolidation (Nadel & Moscovitch, 1997), cerebral GM volume reduction in hippocampus and parahippocampal gyrus might account for memory loss in both svMCI patients and SVaD patients. In addition, superior temporal gyrus is proven to be related with social interaction and cognition process (e.g., visual perception and integration, voice and auditory perception, and even thinking process) (Zilbovicius et al., 2006), which is crucial for the function of language. Thus, cerebral GM volume reduction in superior temporal gyrus might result in language dysfunction in both svMCI patients and SVaD patients. Frontal‐subcortical circuits contain anterior cingulate circuit, dorsolateral prefrontal circuit and orbitofrontal circuit, which mediate many aspects of human behavior and its lesion is related to behavioral disorders such as executive dysfunction, neuropsychiatric symptom, disinhibition, apathy, and depression (Cummings, 1993). Besides the area of cerebral GM volume reduction in svMCI patients, SVaD patients showed more atrophy, predominantly in anterior cingulate, superior frontal gyrus, and orbitofrontal cortex, which could cause disruption of frontal‐subcortical circuits, and consequently account for executive dysfunction, neuropsychiatric symptom, and depression in SVaD patients.

In this study, we have found that both svMCI patients and SVaD patients have larger number of lacunes and WMH volume than healthy controls, indicating that WMH and multiple lacunar infarctions are the characteristics of cerebral small vessel disease in subcortical region. However, no significant difference was found when comparing number of lacunes and WMH volume between svMCI and SVaD patients. Considering the different degree of cognitive impairments between svMCI and SVaD patients, we speculated that subcortical ischemic lesions (e.g., WMH or multiple lacunar infarctions) do not correlate with cognitive impairments directly, which is incompatible with previous researches that subcortical ischemic lesions, such as leukoaraiosis, are associated with cognitive deficits (Kumral et al., 2015; Lamar et al., 2011). According to the conclusion in our study that the cerebral GM volume reduction in both svMCI patients and SVaD patients correlates with cognitive impairments, we speculate that it is cerebral GM volume reduction that results in cognitive impairments, rather than subcortical ischemic lesions. However, the mechanism leading to cerebral GM volume reduction in svMCI patients and SVaD patients still remains unknown.

One possible explanation is cortical ischemia due to occlusions of microvessels or chronic hypo‐perfusion (Garcia, Lassen, Weiller, Sperling, & Nakagawara, 1996), which is the underlying pathogenesis of cerebral small vessel disease. Long‐term hypo‐perfusion may lead to incomplete infarction or microinfarction in cortical region, which cannot be detected by conventional MRI or CT (Garcia et al., 1996). Transneuronal degenerations may happen subsequently after the damage of axon in brain (Ginsberg & Martin, 2002; Johnson & Cowey, 2000) and result in cortical atrophy and cognitive impairments.

Another possible explanation is that the interruption of cholinergic pathways, caused by subcortical ischemic lesions, may play an important role in cortical atrophy (Liu et al., 2014). Lots of studies have found that svMCI patients and SVaD patients showed cerebral GM volume reduction in perisylvian area (Liu et al., 2014; Seo et al., 2010) (predominantly in insula, inferior frontal gyrus and superior temporal gyrus), which is in accordance with our research. The reason why perisylvian area is the most affected region in svMCI patients and SVaD patients may have something to do with the interruption of cholinergic pathways. The perisylvian branch of lateral cholinergic pathways originate from nucleus of Meynert, and it supply the superior temporal gyrus and insula (Selden, Gitelman, Salamon‐Murayama, Parrish, & Mesulam, 1998), where we have also detected GM volume reduction in svMCI and SVaD patients. The medial cholinergic pathways travel within cingulate, project and transmit acetylcholine to the surrounding cortical area (Selden et al., 1998), and we have also detected significant cerebral GM volume reduction at cingulate and paracingulate gyrus in SVaD patients (including anterior and middle cingulate, as well as medial part of superior frontal gyrus). In addition, our study found SVaD patients presented cerebral GM volume reduction in orbitofrontal cortex, which is the place that the medial and lateral cholinergic pathways merge together anteriorly (Selden et al., 1998).

## 5 LIMITATIONS

Some researchers have used 11C‐Pittsburgh compound B (PiB) PET imaging technique to find amyloid deposition in SVaD patients, which could cause cognitive decline independently (Ye et al., 2015). Due to conditional restriction, we did not use PiB‐PET imaging technique to exclude patients with mixed dementia, although we excluded patients whose HIS score<7.

## 6 CONCLUSION

Above all, although there are some limitations, we can still bring forth the conclusion that both svMCI patients and SVaD patients exhibit cerebral GM volume reduction and there exist a hierarchy between svMCI and SVaD, and cerebral GM volume reduction in both svMCI patients and SVaD patients correlates with cognitive deficits, which can help us understand the mechanism of cognitive impairments in svMCI patients and SVaD patients, and diagnose SVaD at its early stage.

## CONFLICT OF INTEREST

None declared.
